# Protective Effect of Triptolide against Glomerular Mesangial Cell Proliferation and Glomerular Fibrosis in Rats Involves the TGF-***β***1/Smad Signaling Pathway

**DOI:** 10.1155/2015/814089

**Published:** 2015-09-14

**Authors:** Yingjie Cao, Xinzhong Huang, Yaping Fan, Xiaolan Chen

**Affiliations:** Department of Nephrology, The Affiliated Hospital of Nantong University, Nantong, Jiangsu 226000, China

## Abstract

Triptolide as a main active ingredient of *Tripterygium wilfordii* is known to be exerting anti-inflammatory, marked immunosuppressive, and podocyte-protective effects. In this study, we investigated the protective effect of triptolide in kidney disease. Rat glomerular mesangial cells were randomly divided into three groups: (1) control group, (2) TGF-*β*1 (10 *μ*g/mL) group, and (3) triptolide group (triptolide 10 *μ*g/L + TGF-*β*1 10 *μ*g/L). Sixty male Sprague-Dawley rats were randomly divided into three groups: (1) control group, (2) chronic serum sickness glomerulonephritis model group, and (3) triptolide (0.2 mg/kg·d) group. Reverse transcription PCR was used to assess Ski and Smad3 mRNA expression in the mesangial cells and renal tissues. Western blotting was used to determine Ski and Smad3 protein expressions. Laser confocal fluorescence microscopy was used to observe the subcellular localization of Smad3 and Ski proteins in the mesangial cells. Triptolide inhibited the TGF-*β*1-induced proliferation of mesangial cells. It significantly upregulated Ski protein expression and downregulated Smad3 mRNA and protein expressions in a time-dependent manner. Laser confocal fluorescence microscopy detected high Smad3 fluorescence intensity in the cytoplasm and low Smad3 and high Ski fluorescence intensity in the nucleus. By upregulating Ski protein expression triptolide decreased the extent of fibrosis by affecting the TGF-*β*1/Smad3 signaling pathway.

## 1. Introduction

Transforming growth factor-beta 1 (TGF-*β*1) is an important regulator of renal fibrosis and TGF-*β*1 gene activation, while its corresponding signaling protein Smad plays an important role in fibrosis in other organs and tissues [1]. TGF-*β*1 promotes the proliferation of mesangial cells, and the TGF-*β*1/Smad3 signal transduction pathway participates in renal fibrosis [2]. There is a very close relationship between the TGF-*β*1/Smad3 pathway and the Ski-SnoN protein family. TGF-*β*1 regulation is influenced by the expression of its receptor genes, the extent of its activation, and other factors such as the signal transduction pathway involving Smad when TGF-*β*1 combines with its corresponding receptors. Studies show that, upon interaction with Smad3, Ski inhibits Smad3-mediated genetic transcription, while, in other cell types, Ski inhibits the TGF-*β* signaling pathway by interacting with Smad3 [3, 4]. Few studies have investigated the roles of Ski and the TGF-*β*1/Smad3 signaling pathway in renal fibrosis.

Extracts of* Tripterygium wilfordii* Hook F (TwHF) have been used in the treatment of glomerulonephritis in China. Triptolide, one of the main active ingredients of extracts of TwHF, has pharmacological effects such as inflammatory response inhibition and immune suppression, antitumor and antifertility activities, direct inhibition of the fusion of podocyte foot processes in Heymann nephritis, and upregulation of key molecules in the podocyte slit diaphragm such as nephrin and podocin. Triptolide showed a prominent antialbuminuric effect in DN. This effect was characterized by an improvement in foot process effacement and the recovery of podocyte injury markers, nephrin and podocin [5–9]. The protective effect of triptolide on podocytes has been well studied, but its role in the inhibition of mesangial cell proliferation and prolongation of renal fibrosis requires further research [10, 11]. In the present study, we investigated the effects of triptolide on renal fibrosis via in vitro cell culture and animal models in an attempt to elucidate the pathogenesis of chronic kidney disease and renal fibrosis and facilitate the formulation of new strategies for the treatment and management of renal fibrosis.

## 2. Materials and Methods

### 2.1. Materials

In this study, we used the following materials: RPMI 1640 powdered cell culture medium (Gibco); fetal bovine serum (FBS; HyClone); recombinant human TGF-*β*1 (PeproTech); MTT kit, Trizol kit, total protein extraction solution, phenylmethylsulfonyl fluoride, immunoprecipitation (IP) cell lysate, and Prestained Dual-Color Protein Molecular Weight Marker (Haimen Bi Yuntian, Jiangsu); a reverse transcription (RT) polymerase chain reaction (PCR) kit (Bioer, Hangzhou); SYBR Premix Ex Taq, RT kit, fluorescence quantitative PCR kit, and dNTPs (TaKaRa, Japan); Ski and Smad3 primers (Shanghai Sangon); *β*-actin, Ski, Smad3, TGF-*β*1, and rabbit anti-collagen type I monoclonal antibodies (Abcam); DyLight 800-labeled mouse IgG antibody and Odyssey infrared laser imaging system (LI-COR); fluoroisothiocyanate- (FITC-) labeled goat anti-mouse IgG antibody (Santa Cruz); Hypersensitive Ready-to-Use Two-Step Detection kit (secondary antibodies; Zhongshan, Beijing); total RNA extraction solution (Biozol, Hangzhou); triptolide (Zelang, Nanjing) (the purity of triptolide, detected by high-performance liquid chromatography, was 99%); enzyme-linked immunosorbent assay microplate reader (BioTek); real-time quantitative PCR instrument (ABI); and laser scanning confocal microscope (Leica, Germany).

### 2.2. Animals

Sixty healthy, 5-week-old, male Sprague-Dawley (SD) rats, weighing 200 ± 20 g, were provided by the Experimental Animal Center of Nantong University and were maintained using conventional feeding. All animal experiments were conducted with the approval of the ethics committee of Nantong University and in accordance with the guidelines for the care and use of laboratory animals in research.

### 2.3. Experimental Methods

#### 2.3.1. Cell Culture, Animal Models, and Grouping

20 mg of triptolide would be dissolved in 2 mL of DMSO to prepare a storage solution with a concentration of 10 mg/mL and stored in a −20°C refrigerator. The culture medium would be diluted to the required concentration prior to usage, with the DMSO concentration not exceeding 0.01‰.

Rat mesangial cells (HBZY-1; American Type Culture Collection) were cultured for 24 h in RPMI 1640 medium supplemented with 10% FBS at 37°C under 5% CO_2_. The samples were then randomly separated into three groups: (1) normal control group, (2)* TGF-β1* group (10 *μ*g/L), and (3) triptolide group (10 *μ*g/L triptolide + 10 *μ*g/L* TGF-β1*) [7–10]. The SD rats were also randomly divided into three groups of 20 rats each: (1) normal control group, (2) chronic serum sickness glomerulonephritis model group, and (3) triptolide group. The rats were adaptively fed for a week. They were then anesthetized with 10% chloral hydrate, and their left kidneys were resected. One week later, a solution of 3 mg bovine serum albumin (BSA) mixed with complete Freund's adjuvant solution (according to the volume ratio of 1 : 1) was injected in the footpad of each rat; this procedure was repeated after every 2 weeks. Two weeks after the first BSA footpad injection, the rats were fed 6 mmol/L HCl acidified water containing 0.1% BSA. After three BSA injections, blood samples were collected from the inner canthus, and the serum anti-BSA antibody titers were detected using double immunodiffusion every day. When the antibody titers reached l : 16, intraperitoneal injections of 3 mg BSA were administered daily to the rats. Three weeks later, an intraperitoneal injection of 100 *μ*g lipopolysaccharide was administered to the rats. At 4 weeks after this, the rats developed proteinuria and decreased renal function. Under light microscopy, diffuse hyperplasia was observed in the glomerular mesangial cells and matrix, indicating that the chronic serum sickness glomerulonephritis model had been successfully established.

The treatment group rats received daily gavages with triptolide (0.2 mg/kg·d) [11], while the rats of model group and control group were administered the same amount of physiological saline. At 7, 14, 21, and 28 days after removal of the left kidneys, 5 rats from each group were killed under intraperitoneal anesthesia, and blood was collected for the measurement of serum creatinine level, blood urea nitrogen (BUN) level, and other biochemical indicators. Simultaneously, the right kidney of the rats was resected. Half of the renal tissue was fixed in 10% formalin solution, embedded in paraffin, and then sliced. The other half was preserved in a refrigerator at −80°C for further use.

#### 2.3.2. MTT Assays for the Detection of Cell Proliferation

Mesangial cells (3 to 10 generations) were plated at a density of 5 × 10^4^/well in 96-well plates, and 200 *μ*L of the RPMI 1640 culture medium was added to each well, with the result being the average value of six additional wells in each group. When the cells had adhered to the walls of the culture vessel, different concentrations (0.4, 2, and 10 *μ*g/L) [7–10] of triptolide were added to the wells. The control group was treated with a cell culture medium not containing triptolide, DMSO, or TGF-*β*1. Three hours later, the cells were separated into groups, and TGF-*β*1 (10 *μ*g/L) was added and allowed to react with the cells for 0, 24, 48, and 72 h, respectively. When the respective target time points were reached, cell culture was stopped, and 5 g/L MTT was added to the mixture, which was then incubated for 4 h at 37°C. The culture medium was then replaced and 150 *μ*L dimethyl sulfoxide was added. The whole mixture was shaken for 10 min to ensure a complete reaction. The absorbance value (*A*) was measured at a wavelength of 570 nm. According to the results of the experiment, we selected the appropriate concentration of triptolide for the next phase of the experiment.

#### 2.3.3. Real-Time Quantitative PCR Assays of TGF-*β*1, Ski, and Smad3 mRNA Expression in Mesangial Cells

Total RNA was extracted from the fourth generation of cells in the fusion state by using the Trizol reagent, and then mRNA was reverse-transcribed to cDNA according to the protocol in the BioRT Two-Step RT-PCR kit. The PCR volume was 20 *μ*L, and the amplification conditions were as follows: predegeneration for 3 min at 95°C; 40 cycles of 95°C for 10 s, 60°C for 30 s, and 72°C for 40 s; 95°C for 15 s; 60°C for 15 s; and finally 95°C for 15 s. After obtaining the cycle threshold, that is, the CT value, the experiment was repeated three times to minimize experimentation errors. The CT values of the housekeeping genes were subtracted from those of the target genes to obtain the CT value; then, ΔCT of the different groups was subtracted from ΔCT of the normal group to obtain ΔΔCT. Thus, by means of 2-ΔΔCT analysis method, the difference in mRNA expression between the experimental and control groups was assessed and analyzed.

All samples for each gene were run in duplicate. Gene expression values were calculated using the comparative threshold cycle (Ct) method, normalized to the expression values of *β*-actin, and displayed as fold induction relative to control. The experiment was repeated three times.

#### 2.3.4. Western Blot Analysis of Ski and Smad3 Protein Expression in Mesangial Cells

The cells were treated as mentioned above, and then IP cell lysate was added to them. This mixture was kept in an ice bath for 30 min, subjected to high-speed centrifugation for 15 min, and the supernatant was retrieved. The bicinchoninic acid (BCA) assay was used to measure the protein concentration in the supernatant. A 5x sample-buffer mixture was boiled for 5 min, and gel electrophoresis was performed. Dual-Color Protein Molecular Weight Marker was added to the protein samples in each running lane, and sodium dodecyl sulfate polyacrylamide gel electrophoresis (SDS-PAGE) was performed, followed by film transfer. The membrane was then incubated overnight with mouse *β*-actin (1 : 4000) and Ski and Smad3 (1 : 200) monoclonal antibodies at 4°C. The following day, the membrane was washed with Tris-buffered saline with Tween20 (TBST). DyLight 800-labeled mouse and rabbit IgG antibodies (1 : 4000) were added to the membrane, which was then incubated for 2 h. The Odyssey infrared laser imaging system was used to scan and analyze the film, and the ratios of the Ski and Smad3 bands with the *β*-actin protein band were used to perform semiquantitative analyses. The experiment was repeated three times and the statistical mean was used to eliminate the scanning and analytic errors.

#### 2.3.5. Laser Confocal Fluorescence Microscopy for the Subcellular Localization of Ski and Smad3 Proteins in Mesangial Cells

The above membranes were fixed in 4% paraformaldehyde for 30 min and were ruptured by reacting them with 0.2% Triton X-100 for 15 min. Then, 5% BSA was added to the membrane, which was enclosed for 30 min. Monoclonal antibodies of Ski and Smad3 (1 : 200) were added to the mixture, which was incubated overnight at 4°C (0.01 mol/L phosphate-buffered saline instead of the primary antibody was used as a negative control). FITC-labeled goat anti-mouse IgG antibody (1 : 4000) was added to the mixture, which was then incubated for 2 h in the dark at room temperature. The mixture was further incubated with Hoechst solution for 5 min at room temperature and then mounted in Tris-buffered glycerol solution. Finally, the membrane was mounted in Tris solution at 4°C in the dark, and, eventually, the subcellular localization of the proteins was determined using confocal laser scanning microscopy. The excitation wavelengths of the laser were 488 nm and 380 nm.

#### 2.3.6. Real-Time Quantitative PCR Assay of TGF-*β*1, Ski, and Smad3 mRNA Expression in Renal Tissues

Total RNA was extracted from the kidney tissues and subjected to fluorescence quantitative PCR assays; cDNA was then synthesized using RT. With the cDNA as a template, PCR was performed using the SYBR@ PrimeScript RT-PCR kit II, according to the manufacturer's instructions. The required primers were synthesized by the Shanghai Biological Engineering Company (Table 1).

The PCR conditions were as follows: predegeneration at 94°C for 30 s, followed by 35 cycles of 61°C for 30 s and 72°C for 30 s. The real-time PCR instrument ABI 7500 was used to analyze TGF-*β*1, Ski, and Smad3 gene expression in each group. At the end of each cycle, the real-time fluorescence value was measured, and, at the end of all cycles, the melting curve was determined. The cycle threshold (CT value) of each sample was determined using ABI 7500 software V2.0.4, and gene expression was analyzed using relative gene quantification.

#### 2.3.7. Western Blot Analysis of Ski, TGF-*β*1, and Smad3 Protein Expression in Renal Tissues

Protein extracting solution was added to approximately 200 g of frozen renal cortex sample, and the mixture was fully ground, transferred to a cold centrifuge tube, and centrifuged for 10 min at 3000 R/min and 4°C. The supernatant, which would be cytoplasmic protein, was transferred to a new Eppendorf tube. The protein concentration was then measured by the BCA method, and after boiling a 5x sample-buffer mixture for 5 min, gel electrophoresis was performed. Protein marker was added to the samples in each lane, and SDS-PAGE was performed. After the electrophoresis, the sample was transferred to a new membrane, and mouse *β*-actin, TGF-*β*1, Ski, and Smad3 monoclonal antibodies were added to the sealed system, which was then incubated overnight at 4°C. The following day, the membrane was washed with TBST; mouse anti-rabbit IgG monoclonal antibody (1 : 4000) was then added, and the membrane was further incubated for 2 h. The Odyssey infrared imaging system with laser scanning was used to scan the results, which were analyzed using GISl000 software. This software attributed each specific data point a gray value, which enabled the semiquantitative analysis of protein expression.

#### 2.3.8. Statistical Analysis

SPSS ver. 15.0 (SPSS, Chicago, IL, USA) was used to perform the statistical analyses. Data were expressed as mean ± standard deviation. Statistical significance was determined using one-way analysis of variance. Differences with *P* < 0.05 were considered statistically significant.

## 3. Results

### 3.1. Effect of Triptolide on TGF-*β*1-Induced Mesangial Cell Proliferation

Compared to the normal control group, the TGF-*β*1-treated group showed significantly greater proliferation at each time point (*P* < 0.05). The addition of triptolide inhibited this effect in a dosage- and time-dependent manner. Cell proliferation was significantly lower after treatment with different concentrations of triptolide for different time points than that observed in the TGF-*β*1 group (*P* < 0.05). This effect of triptolide gradually increased over a 48-h period, peaking at 48 h, and eventually decreasing after 48 h. When the triptolide concentration was gradually increased from 0.4 to 10 *μ*g/L, the efficiency of the drug simultaneously increased, and cell proliferation was most obviously inhibited after treatment with 10 g/L triptolide for 48 h.

**Figure 1:**
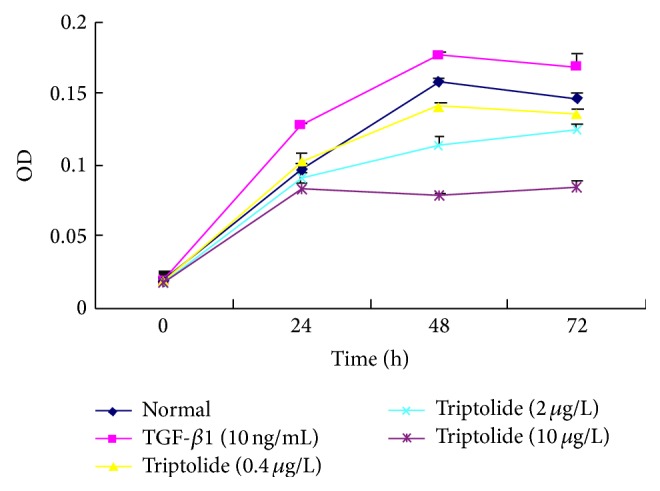
The effect of triptolide on the TGF-*β*1-induced RMC proliferation. Triptolide inhibited the TGF-*β*1-induced proliferation of mesangial cell. The triptolide effect enhanced gradually over a period of 48 h reaching its peak at 48 h and eventually decreasing after 48 h. When the concentration of the triptolide was gradually increased from 0.4 to 10 *μ*g/L, the efficiency of the drug simultaneously increased (*P* < 0.05, *n* = 6).

The cell proliferation in the different groups treated with different concentrations of triptolide at different time points was significantly lower than that in the TGF-*β*1 group (*P* < 0.05; Figure 1).

### 3.2. Effect of Triptolide on Ski and Smad3 Expression in TGF-*β*1-Treated Mesangial Cells

**Figure 2:**
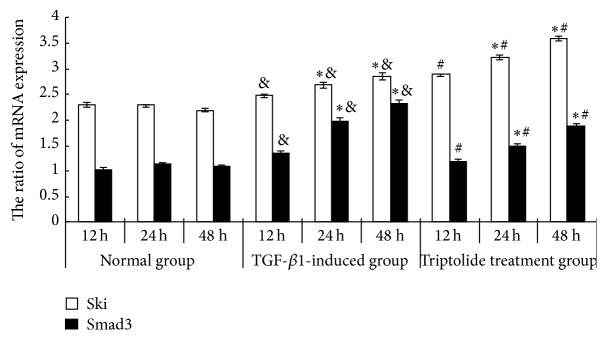
Effect of triptolide on the mRNA expression of Ski and Smad3 of the TGF-*β*1-induced mesangial cells. Triptolide downregulated Smad3 mRNA expression but upregulated Ski mRNA expression in the TGF-*β*1-treated mesangial cells. Compared to the TGF-*β*1-induced group, under the effect of triptolide, the level of expression of Smad3 mRNA decreased but the level of expression of Ski mRNA had significantly increased in a time-dependent manner (^&^ *P* < 0.05 versus the control group, ^*∗*^ *P* < 0.05 versus the different time points in the same group, and ^#^ *P* < 0.05 versus the TGF-*β*1-induced group, *n* = 6).

**Figure 3:**
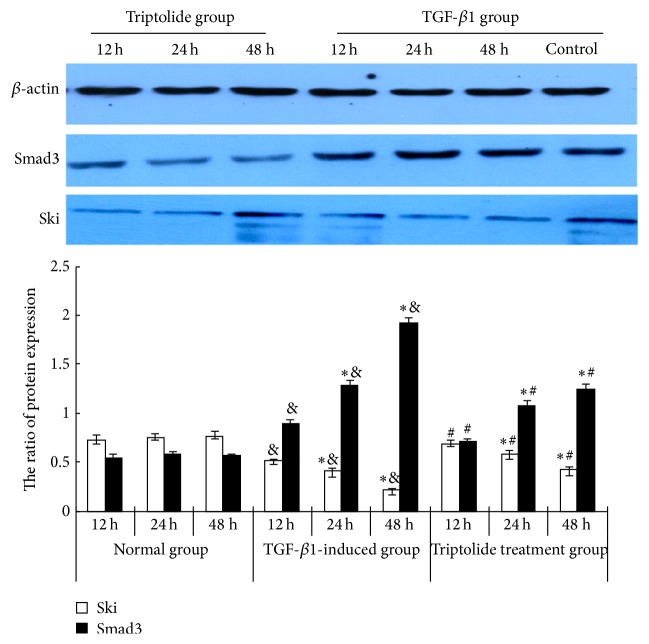
Effect of triptolide on the Ski and Smad3 protein expression of the TGF-*β*1-induced mesangial cells. Triptolide downregulated Smad3 protein expression but upregulated Ski protein expression in the TGF-*β*1-treated cells. Western blot analysis: the protein levels of Smad3 and Ski of the glomerular mesangial cells at different time points in control, TGF-*β*1-stimulated, and triptolide treatment groups. Compared to the TGF-*β*1-induced group, under the effect of triptolide, the level of expression of Smad3 protein decreased but the level of expression of Ski protein had significantly increased in a time-dependent manner (^&^ *P* < 0.05 versus the control group, ^*∗*^ *P* < 0.05 versus the different time points in the same group, and ^#^ *P* < 0.05 versus the TGF-*β*1-induced group, *n* = 6).

The levels of Ski and Smad3 mRNA expression were higher in the TGF-*β*1 group than in the control group (*P* < 0.05). Triptolide downregulated Smad3 mRNA expression but upregulated Ski mRNA expression in the TGF-*β*1-treated mesangial cells in a time-dependent manner (*P* < 0.05; Figure 2). The exposure of the rat mesangial cells to TGF-*β*1 (10 *μ*g/L, 24 h) significantly increased Smad3 protein expression but decreased Ski protein expression, compared with the expression in the control group. Triptolide downregulated Smad3 protein expression but upregulated Ski protein expression in the TGF-*β*1-treated cells in a time-dependent manner (Figure 3).

### 3.3. Effect of Triptolide on the Intracellular Localization of the Ski and Smad3 Proteins in TGF-*β*1-Treated Mesangial Cells

**Figure 4:**
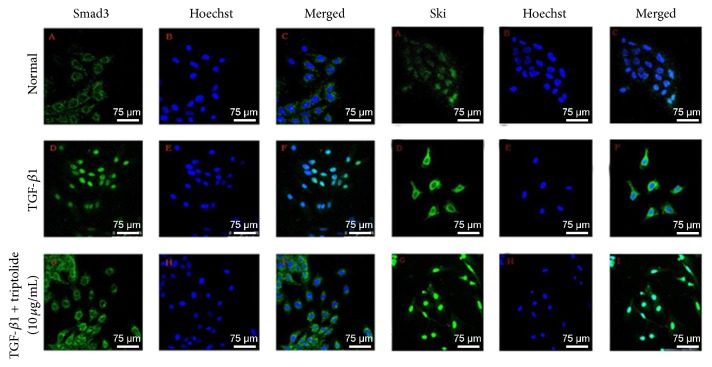
The effect of triptolide on the subcellular localization of the Smad3 and Ski proteins of the TGF-*β*1-induced RMC. In control group, Smad3 protein was mainly expressed in the cytoplasm but, in the TGF-*β*1-induced group, the level of expression of Smad3 protein was significantly higher and was mainly localized in the nucleus. Ski protein of the control group was expressed mainly in the nucleus. The level of expression of Ski in and out of the nucleus of the TGF-*β*1-induced group was significantly higher and was mainly localized in the cytoplasm (bar = 75 *μ*m).

TGF-*β*1 increased Smad3 in the nucleus, while triptolide caused Smad3 to be localized only to the cytoplasm. TGF-*β*1 increased Ski in the cytoplasm, while triptolide increased it in the nucleus (Figure 4).

### 3.4. Changes in Biochemical Indicators in Different Animal Groups

Compared to the control group, the model group showed higher 24-h urine protein, serum creatinine, and BUN levels (*P* < 0.05). In the triptolide group, the levels of the abovementioned biochemical indicators were lower than those in the model group (*P* < 0.05; Table 2).

### 3.5. Pathological Changes in Renal Tissues

**Figure 5:**
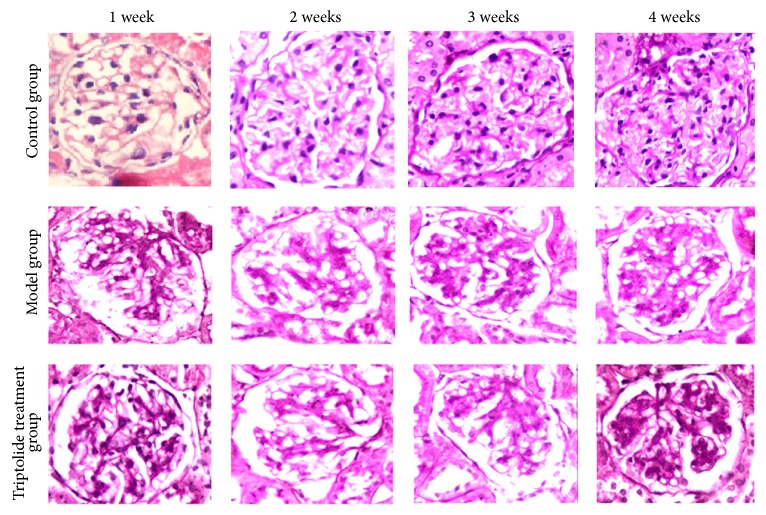
Renal pathological changes of the different groups at different time points. Triptolide alleviated pathological damage of rat renal tissues in chronic serum sickness glomerulonephritis model group. There was no significant hyperplasia in the glomerular mesangial cells and cellular matrix, with no obvious broadening of the mesangial region in the renal tissues from the normal control group. However, there was significant hyperplasia in the glomerular mesangial cells and cellular matrix, with significant broadening of the mesangial region in the renal tissues obtained from the model group, increasing with respect to time, with the tendency to glomerular fibrosis. The lesions of the triptolide treatment group were, however, alleviated.

Light microscopy revealed that the model group showed significant hyperplasia of the glomerular mesangial cells and increased matrix deposition, indicating that the model was successfully established. In the triptolide group, these changes were comparatively alleviated (Figure 5).

### 3.6. Effect of Triptolide on Ski and Smad3 Expression in Renal Tissues of Different Animal Groups

**Figure 6:**
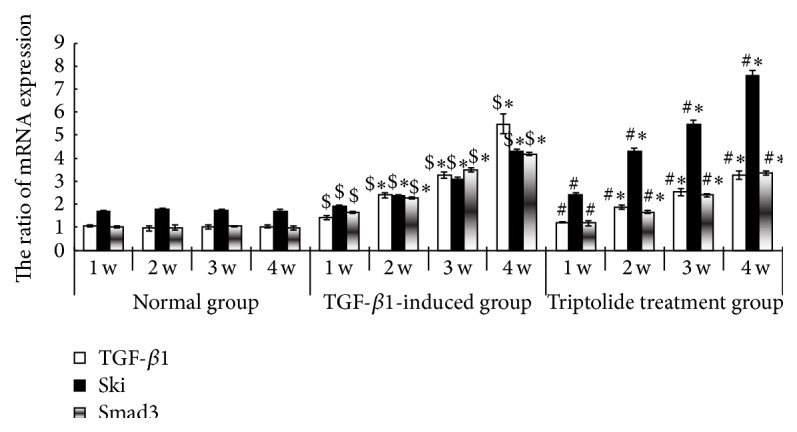
The expression of TGF-*β*1 mRNA, Smad3, and Ski in the renal tissues of the different groups. Triptolide downregulated Smad3 mRNA expression but upregulated Ski mRNA expression in the renal tissues of the model rats. In the model group, the level of expression of TGF-*β*1 and Smad3 mRNA in the renal tissues of the rats of the model group at the different time points was high and time-dependent but the expression of Ski mRNA was lower than that in the control group. Compared to the triptolide treatment group, the level of expression of TGF-*β*1 and Smad3 mRNA in the renal tissues of the rats of the model group at the different time points was lower and time-dependent but the expression of Ski mRNA was higher (^$^ *P* < 0.05 versus the control group, ^*∗*^ *P* < 0.05 versus the different time points in the same group, and ^#^ *P* < 0.05 versus the TGF-*β*1-induced group, *n* = 18–20).

**Figure 7:**
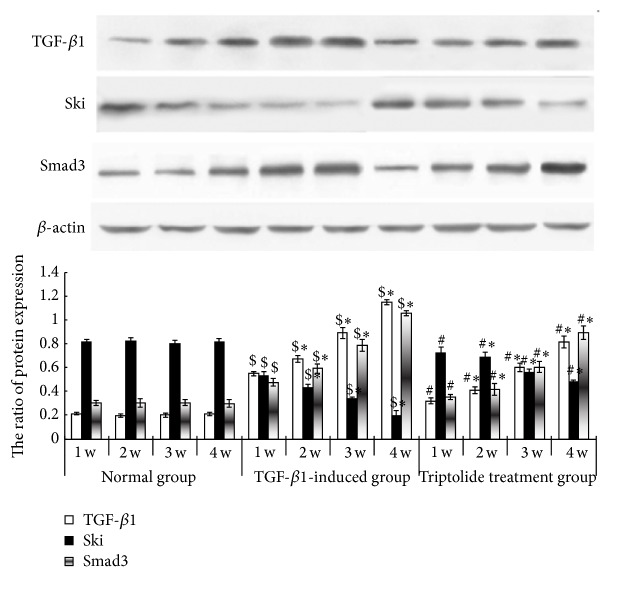
Expressions of TGF-*β*1, Smad3, and Ski proteins in the renal tissues of the different groups. Triptolide downregulated Smad3 protein expression but upregulated Ski protein expression in the renal tissues of the model rats. Western blot analysis of Ski, TGF-*β*1, and Smad3 protein expressions in the renal tissues at different time points in the control, model, and triptolide treatment groups. In the model group, the level of expression of TGF-*β*1 and Smad3 proteins in the renal tissues of the rats of the model group at the different time points was higher and time-dependent but the expression of Ski protein was lower than that in the control group. Compared to the triptolide treatment group, the level of expression of TGF-*β*1 and Smad3 protein in the renal tissues of the rats of the model group at the different time points was lower and time-dependent but the expression of Ski protein was higher (^$^ *P* < 0.05 versus the control group, ^*∗*^ *P* < 0.05 versus the different time points in the same group, and ^#^ *P* < 0.05 versus the TGF-*β*1-induced group, *n* = 18–20).

Compared to the control group, the model group showed increased TGF-*β*1, Smad3, and Ski mRNA expression in the renal tissues, and this increase was time-dependent (*P* < 0.05). After triptolide treatment, TGF-*β*1 and Smad3 mRNA expression was significantly decreased and Ski mRNA expression was increased, as compared to the levels in the model group (*P* < 0.05; Figure 6). Compared to the control group, the model group showed increased TGF-*β*1 and Smad3 protein expression in the renal tissues, and the expression increased in a time-dependent manner; in contrast, Ski protein expression was lower in the model group than in the control group (*P* < 0.05). After triptolide treatment, TGF-*β*1 and Smad3 protein expression was significantly decreased and Ski protein expression was increased, compared to the levels in the model group (*P* < 0.05; Figure 7).

## 4. Discussion

TGF-*β*1 is an important fibrogenic factor involved in the occurrence and development of glomerular sclerosis. Qu et al. and Zhu et al. demonstrated that Smad protein is currently the only known intracellular kinase substrate of the TGF-*β*1 receptor. Smad proteins are divided into three types; however, only Smad2, Smad3, and Smad7 participate in the signal transduction of TGF-*β*1. The combination of the Smad protein and a transcriptional coactivator causes the upregulation of TGF-*β*1 transcription-targeted genes [9, 10]. Many studies have shown that excessive activation of the TGF-*β*/Smad3 signaling pathway can promote the progression of renal fibrosis. On the other hand, the combination of the Smad protein with transcriptional corepressors, including Ski protein and SnoN protein (which resembles Ski protein), can inhibit signal transduction via the TGF-*β*1 signaling pathway [11–15]. Zheng et al. and Nishida et al. demonstrated that Ski is a negative regulatory factor in the TGF-*β*1/Smad signaling pathway; through its interaction with the Smad3 protein, Ski inhibits the activation of the target genes of TGF-*β*1, thereby regulating the TGF-*β*1/Smad signaling pathway [16–18]. Cheng et al. demonstrated that, in normal cells, Ski is expressed mainly in the nucleus and rarely in the cytoplasm. It has DNA-binding activity and is degraded mainly through the ubiquitin proteasome pathway [19].

Triptolide (*Tripterygium wilfordii* Hook F) is one kind of* Euonymus alatus* plant and is presently one of the traditional Chinese medicines with obvious immunosuppressive properties. Multiglycoside of* Tripterygium wilfordii* Hook F, GTW, the crude extract of the triptolide root, has anti-inflammatory, immunosuppressive, antifertility, and anticancer properties. The main active component of multiglycoside of* Tripterygium wilfordii* Hook F is triptolide (TP).

Triptolide and its derived compound (5R)-5-hydroxytriptolide (LLDT-8) are insoluble in water; hence, their metabolism in vivo mainly depends on the cytochrome P450 enzyme function [16, 20]. The challenges prevailing in research work about triptolide and its clinical application in China are illustrated as follows: the sample size is too small, there is no clear or precise randomization, there is no application of blinding or masking research techniques, the event outcome is not complete, and so on. These shortcomings have caused the lack of high quality clinical research works, despite the wide application of triptolide in China, leading to the lack of high level evidence, making it difficult to set up a proper protocol. Zhu et al. demonstrated that triptolide inhibits extracellular matrix protein synthesis by suppressing Smad2 [10]. Chen et al. demonstrated that triptolide inhibits the TGF-*β*1-induced proliferation of rat airway smooth muscle cells by suppressing Smad3 signaling [20].

In the present study, we performed in vitro cultures of mesangial cells and observed that TGF-*β*1 induced significant mesangial cell proliferation. In addition, TGF-*β*1 inhibited Smad3 expression at the gene and protein levels in a dosage-dependent manner.

To verify these findings, we performed in vitro research and established the chronic serum sickness glomerulonephritis rat model. While setting up the model, we discovered that the hyperplasia of glomerular mesangial cells and cellular matrix and the levels of clinical biochemical indicators, such as 24-h urine proteins and serum creatinine, displayed an increasing trend, suggesting that the model was successfully set up. After conducting the experiments, we found that the mRNA and protein expressions of TGF-*β*1 and Smad3 were lower and the Ski mRNA and protein expressions were higher in the triptolide group than in the model group. Hence, the results of the animal model and in vitro cell culture were found to be consistent. Further experiments showed that, by upregulating Ski protein expression, triptolide inhibited the proliferation of the mesangial cells and cellular matrix.

We also investigated the subcellular localization of Smad3 and found that Smad3 mainly existed in the cytoplasm of normal mesangial cells; its expression in the nucleus was low in normal cells. TGF-*β*1 promoted the nuclear translocation of Smad3, and triptolide significantly inhibited Smad3 expression, its nuclear translocation, and the transcription of nuclear target genes.

Bonnon and Atanasoski have found that Ski expression in normal mesangial cells is uniform in and out of the nucleus, but the expression level is relatively low [21]. After treatment with TGF-*β*1, Ski expression was increased mainly in the cytoplasm, and this finding could be attributed to feedback regulation. Ski expression was increased after triptolide treatment and was obvious mostly in the nucleus, further suggesting that the decrease in Ski protein expression was not due to the regulation of Ski mRNA levels but rather due to the accelerated degradation of the translated protein via the ubiquitin-dependent proteasome pathway [22–24]. The regulation of Ski is a result of the posttranslation modification of proteins and has nothing to do with the level of transcription. This finding is consistent with related reports [22, 25–29]. There are two possible ways via which Ski can inhibit the TGF-*β*1/Smad signal transduction pathway. (1) As a transcriptional corepressor, Ski inhibits TGF-*β*1-mediated gene translation in the nucleus. (2) Ski binds with Smad3 in the cytoplasm and interferes with its nuclear translocation, thereby inhibiting renal fibrosis.

## 5. Conclusions

In conclusion, the Ski protein, acting as a key regulatory protein, can inhibit TGF-*β*1-mediated fibrosis. Triptolide showed a protective effect against renal fibrosis. The therapeutic effect of triptolide may be attributable to the inhibition of TGF-*β*1 and Smad3 expression and the upregulation of Ski protein expression. Our results suggest that the protective effect of triptolide in renal fibrosis might be related to the inhibition of the TGF-*β*1/Smad signaling pathway.

## Figures and Tables

**Table 1:** The sequence and length of the primers.

Primer	Sequence	Length (kb)
*Ski* forward primer	5′-GAGGATGTYCAGGCAGTGGTGC-3′	169
*Ski* reverse primer	5′-TGGGAAGGATGCAGCTAAAGTG-3′	

*Smad*3 forward primer	5′-CAGGGCTTTGAGGCTGTCTACC-3′	104
*Smad*3 reverse primer	5′-GTGCTGGTCACTGTCTGTCTCCT-3′	

TGF-*β*1 forward primer	5′-CCAACTATTGCTTCAGCTCCA-3′	157
TGF-*β*1 reverse primer	5′-GTGTCCAGGCTCCAAATGT-3′	

*β*-actin forward primer	5′-TTTAATGTCACGCACGATTTC-3′	150
*β*-actin reverse primer	5′-CCCATCTATGAGGGTTACGC-3′	

**Table 2:** Changes in the biochemical indicators of the groups.

Changes in the biochemical indicators of group	Time (week)	24 h urine protein (mg/24 h)	Urine nitrogen (mol/L)	Creatinine (*μ*mol/L)
Control group	1	1.8 ± 0.24	5.38 ± 0.35	33.68 ± 3.24
	2	2.0 ± 0.41	5.32 ± 0.21	32.45 ± 2.65
	3	2.3 ± 0.38	5.49 ± 0.28	31.88 ± 2.56
	4	1.9 ± 0.54	5.56 ± 0.48	35.27 ± 3.08

Model group	1	8.3 ± 0.61^a^	8.38 ± 0.35^a^	43.67 ± 3.05^a^
	2	19.3 ± 3.18^ac^	11.32 ± 1.21^ac^	52.46 ± 3.65^ac^
	3	56.3 ± 10.36^ac^	13.47 ± 2.08^ac^	71.78 ± 4.56^ac^
	4	124.3 ± 13.42^ac^	15.58 ± 3.42^ac^	85.19 ± 6.08^ac^

Triptolide treatment group	1	5.3 ± 0.52^b^	6.39 ± 0.95^b^	39.68 ± 3.12^b^
	2	10.3 ± 2.78^bc^	8.32 ± 1.21^bc^	42.45 ± 3.61^bc^
	3	21.3 ± 5.25^bc^	10.47 ± 1.48^bc^	58.76 ± 4.56^bc^
	4	48.3 ± 11.32^bc^	11.58 ± 1.42^bc^	69.21 ± 4.28^bc^

*n* = 18–20. Note: when compared to the control group, ^a^ *P* < 0.05; when compared to the model group, ^b^ *P* < 0.05; when comparing the values at different time points in the same group, ^c^ *P* < 0.05.
